# The potential of bacteria isolated from ruminal contents of seaweed-eating North Ronaldsay sheep to hydrolyse seaweed components and produce methane by anaerobic digestion *in vitro*

**DOI:** 10.1111/1751-7915.12000

**Published:** 2012-11-22

**Authors:** Allan G Williams, Susan Withers, Alastair D Sutherland

**Affiliations:** Department of Biological and Biomedical Sciences, School of Health and Life Sciences, Glasgow Caledonian UniversityCowcaddens Road, Glasgow, G4 0BA, UK

## Abstract

The production of methane biofuel from seaweeds is limited by the hydrolysis of polysaccharides. The rumen microbiota of seaweed-eating North Ronaldsay sheep was studied for polysaccharidic bacterial isolates degrading brown-seaweed polysaccharides. Only nine isolates out of 65 utilized > 90% of the polysaccharide they were isolated on. The nine isolates (eight *Prevotella* spp. and one *Clostridium butyricum*) utilized whole *Laminaria hyperborea* extract and a range of seaweed polysaccharides, including alginate (seven out of nine isolates), laminarin and carboxymethylcellulose (eight out of nine isolates); while two out of nine isolates additionally hydrolysed fucoidan to some extent. Crude enzyme extracts from three of the isolates studied further had diverse glycosidases and polysaccharidase activities; particularly against laminarin and alginate (two isolates were shown to have alginate lyase activity) and notably fucoidan and carageenan (one isolate). In serial culture rumen microbiota hydrolysed a range of seaweed polysaccharides (fucoidan to a notably lesser degree) and homogenates of *L. hyperborea*, mixed *Fucus* spp. and *Ascophyllum nodosum* to produce methane and acetate. The rumen microbiota and isolates represent potential adjunct organisms or enzymes which may improve hydrolysis of seaweed components and thus improve the efficiency of seaweed anaerobic digestion for methane biofuel production.

## Introduction

Seaweed is an attractive biomass for microbial biofuel production. It does not require irrigation with valuable water resources, it generally has no lignin (which is present in terrestrial plants and is not only difficult to breakdown but reduces degradation, and therefore digestibility, of plant cell wall cellulose complexes), and there is little if any competition for its use as a food source, unlike many terrestrial crops. It can be collected as beach cast seaweed, which is regarded as a pollutant of leisure beaches, or it can be harvested sustainably from natural seaweed beds. It could also be farmed, and it grows rapidly; for example 60 tons dry weight per hectare per year of *Laminaria japonicum* were reported to be produced in China (Morand *et al*., 1991). From the 1970s through to 1990s various groups were involved in assessing the suitability of seaweed (particularly *Macrocystis pyrifera*) as a biomass for anaerobic digestion (AD) to produce methane biofuel (see review by Forro, 1987). The overall result of these studies indicated that seaweeds were generally a suitable biomass for AD.

In the Northern Atlantic *Laminaria*, *Fucus* and *Ascophyllum* species are particularly common brown algae found growing on the litterol and sub-litterol seashore. They are also beach cast in large quantities along the seashore, particularly in western Scotland during the winter (Morand *et al*., 1991). Animal manure is often utilized as an inoculum for anaerobic digesters but various reports have suggested that specific bacteria capable of fermenting marine phycocolloids may accelerate and increase biogas production from seaweeds (Morand *et al*., 1991). Orpin and colleagues (1985) studied the ruminal microbiota of the North Ronaldsay breed of sheep, the male adults of which survive almost entirely on a seaweed diet on the remote island of North Ronaldsay in the Orkney Islands, Scotland. They noted that there were important differences in the speciation and metabolic activity of seaweed-fed North Ronaldsay sheep rumen microbiota compared with grass-fed sheep (particularly, laminarin, mannitol, fucoidan, mannuronic and guluronic acids were far more readily hydrolysed) and concluded that the microbiota adapted to efficiently digest seaweeds. Even in humans, the ability to digest seaweeds has recently been associated with alterations in gut flora; with gut bacteria acquiring seaweed degrading genes from marine species (Hehemann *et al*., 2010).

The efficient hydrolysis of seaweed polysaccharides, particularly alginates, is seen as a rate limiting step for efficient AD to proceed (Morand *et al*., 1991; Horn, 2000). With a long-term aim of developing and characterizing an optimal inoculum with which to degrade seaweeds by AD, we aimed to examine whether bacterial isolates from the rumen microbiota of North Ronaldsay sheep could produce enzymes to effectively hydrolyse seaweed polysaccharides by the concerted actions of polysaccharide depolymerases and glycoside hydrolases. Small scale serial culture studies with the whole rumen microbiota also determined if seaweed polysaccharides and whole seaweed homogenates could be effectively digested to produce methane and acetate.

## Results

### Isolation of polysaccharide degraders

Pre-enrichment in liquid media was preferred to direct isolation of rumen microbiota as the ruminal contents had been frozen and this may have impacted on the viability of the microbial populations. Growth was observed in enrichment cultures with all of the substrates tested and subsequent counts (log_10_ cfu ml^−1^ enrichment culture) on agar plates containing various substrates were: alginate (10.6–10.9), laminarin (10.5–10.7), fucoidan (7.8–8.6) and cellulose (6.7–8.5).

The enrichment cultures resulted in the isolation of 65 colonies on media containing either laminarin (20), alginate (18), fucoidan (11), cellulosic derivatives (12) or *Laminaria hyperborea* extract (4). These were selected as representative isolates on the basis of distinct morphology. The 65 isolates were subsequently tested for polymer utilization in broth culture and only nine degraded > 90% of their recovery polysaccharide. Isolates enriched and recovered on *L. hyperborea* extract were found unable to degrade individual polysaccharide substrates and in all probability were members of the microbial population that utilized non-polymeric cellular constituents such as the storage polysaccharide mannitol and the products released by the degradative population, and exemplified the rationale for not using seaweed extract as the only primary enrichment isolation substrate for degraders.

### Range of polysaccharide utilization

The ability of the nine isolates to degrade a range of seaweed polysaccharides was then assessed. All nine isolates were able to effectively utilize laminarin while seven utilized alginic acid (Table 1). Only strains L7 and L10 exhibited any substantive activity against fucoidan. However, all strains were capable of utilizing carbohydrate constituents present in the seaweed extract and eight exhibited some activity against carboxymethylcellulose (Table 1).

**Table 1:** Seaweed polysaccharide utilization by degradative ruminal isolates

		Utilization (%)^b^				
		
Isolate	Acetate^a^ μmol ml^−1^	Alginate	Laminarin	CMC	*L. hyperborea* extract	Fucoidan
L7^c^	35.1	80.0	95.2	28.4	52.4	18.6
L8	37.3	66.7	93.5	21.7	19.0	8.5
L10	33.6	nd	94.1	16.7	44.0	20.3
L12	44.0	5.8	58.2	3.3	14.5	1.7
A9	32.5	70.0	93.2	24.2	32.1	1.7
A11	43.3	71.1	93.6	20.8	24.9	8.5
A12	39.1	70.6	92.7	17.5	28.6	nd
A14	34.3	71.1	93.6	20.8	22.6	nd
C8	31.0	71.1	93.7	21.7	29.8	nd

a Acetate production on laminarin-supplemented medium.

b The values given are the mean of duplicate cultures; the between-duplicate culture variation was < 10%.

c Isolation substrate L, laminarin; A, alginate; C, cellulose.

nd, not detected.

All the isolates grew on the *L. hyperborea* extract broth (Table 1) but growth (increased absorbance at 595 nm) only occurred with isolate C8 on the mixed *Fucus* extract and by four strains (A9, A12, A14 and L12) on the *Ascophyllum nodosum* extract (data not shown). Addition of glucose stimulated increased growth of all the isolates in the presence of the three seaweed extracts indicating a lack of readily assimilated substrates rather than growth inhibition by substances in the extract. Measurement of residual carbohydrates confirmed that all isolates, excepting L8, had utilized constituents in the *L. hyperborea* extract, whereas evidence of carbohydrate removal from the other two extracts by any of the isolates was inconclusive.

All isolates formed acetate when grown on laminarin (Table 1) and thus have the potential to degrade seaweed polysaccharides and produce a key metabolite for acetoclastic methanogens.

### Selection and identification of three most active isolates

All of the nine isolates were obligately anaerobic and all but L10 and L12 were Gram-negative rods. Isolate L10 withstood heating at 80°C for 15 min and was a presumptive *Clostridium* sp. Eight of the nine isolates were identified to at least Genus level (isolate A12 was not identifiable) by having a high degree of homology (97–100%) with 16S rRNA genes lodged in the NCBI nucleotide database. The species identification also matched morphological and biochemical studies which gave the same presumptive Genus/speciation assignation. A11 and L7 were *Prevotella* species (100% homology) and L10 was a *Clostridium butyricum*. These are common rumen bacteria, and other isolates (A9, A14, L8 and C8) were similarly *Prevotella* spp. Isolate L12 was *Clostridium botulinum*.

Three isolates chosen for further study were: isolate L7, a *Prevotella* sp., was consistently the most effective of the polysaccharide degraders that could also utilize fucoidan (18.6% degradation); L10, a *C. butyricum*, was an effective laminarin degrader that also utilized fucoidan (20.3% degradation); and A11, a *Prevotella* sp. initially recovered on alginic acid, had degradative profiles similar to several of the other isolates, but was chosen for its acetogenic potential.

### Glycosidase and polysaccharidase activities of isolates

The potential of isolates L7, L10 and A11 to utilize, or be involved in the degradation of other polysaccharide classes (e.g. galactans, mannans, xylans etc.) was determined by monitoring the range of their polysaccharide depolymerase (polysaccharidase) and glycoside hydrolase (glycosidase) activities.

Isolates L7 and A11 were found to have wide-ranging glycosidase activities (Table 2). The activity profile was not markedly affected by the growth substrate indicating that growth on seaweed extract and individual seaweed polysaccharides resulted in the expression of a wide range of activities. There was little or no detectable β-l-fucosidase, β-d-glucuronidase, β-d-galacturonidase, β-d-mannosidase and α-d-xylosidase activities (results not shown). The glycosidase profile of L10 was considerably more restricted, although higher activities were detected after growth on seaweed extract. Polysaccharidases active against α-glucans (amylopectin), β-glucans [laminarin (β1-3), cello-oligosaccharides (β1-4)] and alginate were detected in all the isolates (Table 3). There was an indication that the growth substrate did affect detectable activity profiles. There was no evidence for the formation of galactanase or a fucoidanase by L7 or A11 under the growth conditions examined although low levels were apparently present in L10.

**Table 2:** Glycoside hydrolase activities in isolates grown on polysaccharidic substrates and *L. hyperborea* seaweed extract

	Glycosidase-specific activity^a^					
	
*p*-Nitrophenyl derivative glycosidase substrate	A11-alginate^b^	A11-*L. hyperborea*	L7-laminarin	L7*-L. hyperborea*	L10-laminarin	L10-*L. hyperborea*
Alpha-l-arabinofuranoside	55.1	60.4	34.5	59.8	nd	nd
Beta-d-cellobioside	8.4	8.8	23.5	8.4	nd	nd
Alpha-l-fucoside	21.0	22.0	13.4	19.2	nd	nd
Beta-d-fucoside	4.9	7.5	14.8	7.7	nd	1.8
Alpha-d-galactoside	33.9	29.8	29.9	35.3	20.7	26.8
Beta-d-galactoside	68.8	66.5	35.3	61.2	0.6	45.0
Alpha-d-glucoside	7.9	14.2	12.1	14.1	4.7	14.0
Beta-d-glucoside	30.9	28.0	34.9	36.5	0.2	nd
Alpha-d-maltoside	6.9	20.2	10.9	26	0.2	nd
Alpha-d-mannoside	0.9	3.7	0.8	3.2	nd	nd
Beta-d-xyloside	2.3	1.8	7.6	2.0	nd	nd

a Specific activities expressed as nmol *p*-nitrophenol released mg^−1^ protein in 1 min; nd; not detected.

b Isolates were grown in broth supplemented with a single polysaccharide or *L. hyperborea* extract and the results shown are from single samples of pooled duplicate or triplicate cultures respectively.

**Table 3:** Polysaccharidase activities in isolates grown on *L. hyperborea* extract and isolated polysaccharides

	Specific activity^a^					
	
Enzyme substrate	A11-alginate^b^	A11-*L. hyperborea*	L7-laminarin	L7-*L. hyperborea*	L10-laminarin	L10-*L. hyperborea*
Laminarin	1300	860	3098	1110	4529	567
Alginate	1490	1356	103	1621	nd	57
Fucoidan	nd	nd	nd	nd	9.5	27.1
Carrageenan	4	nd	nd	nd	nd	24
Amylopectin	279	500	207	599	833	296
Cello-oligosaccharides	774	834	1651	983	185	72.6
Xylan	35.3	220	35.7	236	14.7	72.8

a Specific activity expressed as nmol reducing sugar formed mg^−1^ protein in 1 h; nd, not detected.

b Isolates were grown in broth supplemented with a single polysaccharide or *L. hyperborea* extract and the results shown are from single samples of pooled duplicate or triplicate cultures respectively.

Alginic acid depolymerization may take place by glycanase-type reactions in which the glycosidic bond is cleaved directly to generate oligomeric fragments or alternatively the chain cleavage may occur via a lyase type reaction in which degradation products with unsaturated reducing ends are produced and detected by their absorbance at 230 nm (Haugen *et al*., 1990). Absorbance increased concomitantly with alginate breakdown. The increase in absorbance (per mg protein after incubation for 90 min) for enzyme preparations recovered from cells grown on the isolation polymer (A11 alginate, L7 L10 laminarin) or *L. hyperborea extract* was 1.28/1.33, 0.24/1.73 and 0.19/0.19 with enzyme from A11, L7 and L10 respectively.

The activity in A11 was expressed similarly when the organism was grown on either alginate or the alginate-containing seaweed extract. However, there was a pronounced difference in the activity of strain L7 after growth on laminarin or the *Laminaria* extract, where growth in the latter resulted in enzyme induction and increased activity. Only background activities were detected in the non-alginolytic strain L10.

### Sequential culture studies

**Fig. 1:**
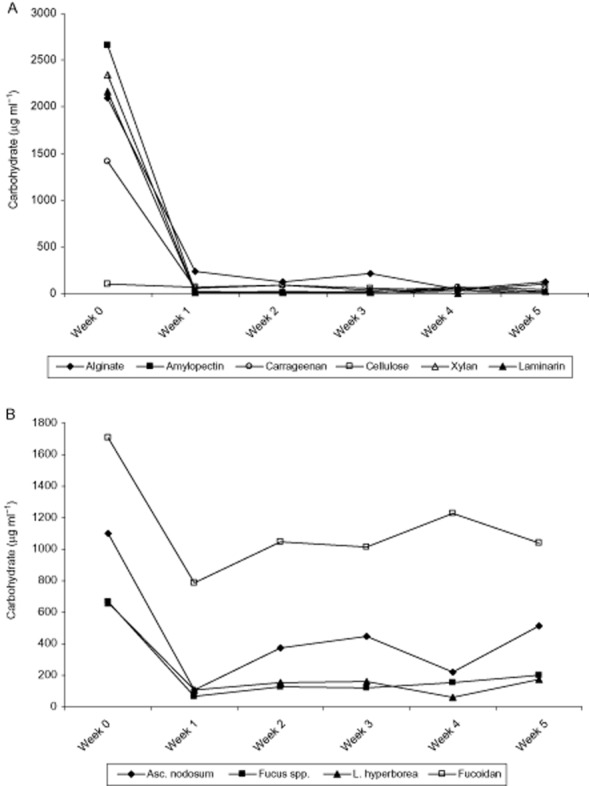
Residual carbohydrate concentrations determined at the end of the 7-day consecutive incubation period for each of the serial cultures. The carbohydrate substrates used were (A) alginate (♦), amylopectin (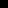), carrageenan (○), cellulose (□), xylan (Δ) and laminarin (▴). In (B) the substrates were fucoidan (□), *Ascophyllum nodosum* (♦), *Fucus* spp. (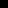) and *Laminaria hyperborea* (▴).

A range of serial cultures of whole North Ronaldsay sheep rumen microbiota were able to utilize seaweed constituents and whole seaweed homogenates as the primary carbohydrate source. The cultures were established by successive serial culture at 7-day intervals over a 6-week period. The isolated polymers were, with the exception of fucoidan, extensively utilized (Fig. 1A and B) and carbohydrate components were also removed from the seaweed homogenates. The efficiency of carbohydrate removal was highest with *L. hyperborea* and lowest from the *Fucus* spp. supplemented culture.

After incubation methane and carbon dioxide was detected in all the serial cultures and with many substrates these were present in proportions normally encountered in the rumen ecosystem, i.e. 30% methane, 65% carbon dioxide.

**Fig. 2:**
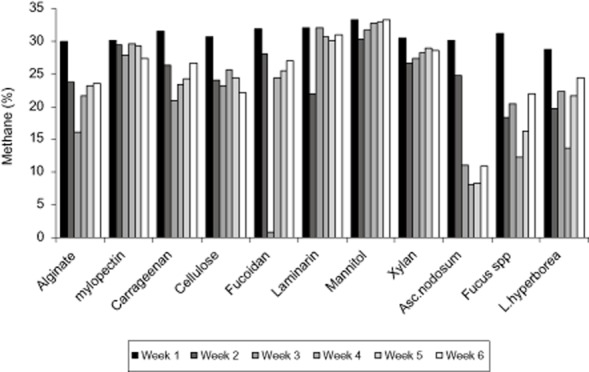
Methane accumulation as monitored at the end of the 7-day consecutive incubation period for each of the serial cultures. The proportion of methane as a percentage of the headspace gas is indicated for various enrichment substrates at the end of week 1 (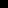) through to the end of week 6 (□).

Stable methanogenic cultures were established with structural polymers and storage polymers including mannitol (Fig. 2) confirming that the ability to catabolize a wide range of seaweed carbohydrates was present in the initial ruminal population. Although fucoidan was catabolized less effectively than other polysaccharides its utilization was also associated with consistent methane formation.

The serial cultures established on seaweed homogenates were also methanogenic, but the proportion of methane detected in the headspace fermentation gases tended to be lower than observed when individual extracted carbohydrates were used (Fig. 2). The methane proportion stabilized at approximately 10% in cultures growing on *A. nodosum* but was of the order of 20–25% in the cultures established on *L. hyperborea* and *Fucus* species (Fig. 2).

**Fig. 3:**
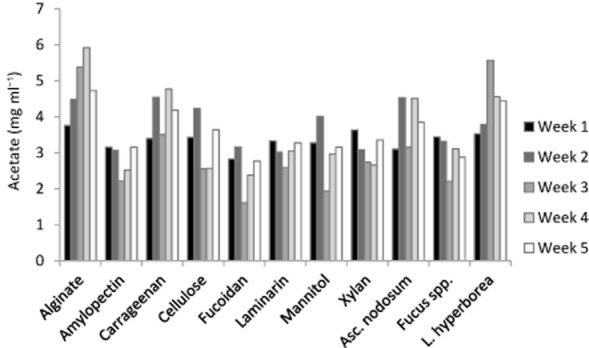
Acetate accumulation as monitored at the end of the 7-day consecutive incubation period for each of the serial cultures. The acetate level is indicated for various enrichment substrates at the end of week 1 (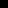) through to the end of week 5 (□).

It was evident (Fig. 3) that all cultures were acetogenic and accumulated acetate during each 7-day culture period. Acetate levels were consistent over the period but were substrate-dependent as fucoidan, which was not extensively utilized, was the least acetogenic. However, seaweed homogenates were as effective substrates for acetate formation as individual polysaccharides (Fig. 3).

## Discussion

In this study the structural integrity of the cellulosic substrate (filter paper) was lost after enrichment culture indicating that cellulolytic activity was present. It is possible that cellulolytic bacteria are only present in low numbers in the North Ronaldsay sheep rumen and this may account for the inability of Orpin and colleagues (1985) to isolate these directly. Orpin and colleagues (1985) also found only 13% of the culturable bacteria from seaweed-fed sheep grew on alginate, 71% on laminarin, 13% on fucoidan and 99% on mannitol. These were however significantly higher percentages than those from pasture-fed animals (2%, 32%, 0% and 0% respectively). Additionally, Orpin and colleagues (1985) found substantial numbers of seaweed-fed rumen bacteria utilized mannuronic and guluronic acids, which are likely to be released on hydrolysis of alginate in the rumen.

In the present study the majority of the 65 isolates recovered from enrichment cultures were found to be unable to degrade polymeric substrates. Many other anaerobic bacteria are reported to grow only when part of a consortium in which mutualistic metabolic exchanges occur (Stams and Plugge, 2009; Hillesland and Stahl, 2010; Miller *et al*., 2010; Summer *et al*., 2011) and many of these isolates may require mutualistic interactions for growth on polymeric substrates.

It was probable, however, that these isolates were effective scavengers of polysaccharide degradation products and soluble sugars and were therefore likely to be important in the microbial consortia in the removal of soluble sugars and generation of methanogenic precursors. It would be worthwhile to screen these non-degrader strains for their capacity to produce acetate.

The ability of nine selected polysaccharide degrading isolates to degrade a range of seaweed polysaccharides was assessed in broth culture. There was only very limited growth in fucoidan and seaweed-extract containing media. This agrees with the findings of Orpin and colleagues (1985), who found few isolates utilized fucoidan and none in pasture fed animals. Isolates that degrade this sulfated polysaccharide (such as L7 and L10) are then potentially important.

The wide ranging glycosidase activity profiles of isolates L7 and A11 are compatible with them being strains of *Prevotella* (formerly *Bacteroides*) *ruminicola* as studies by Williams and colleagues (1984) demonstrated similarly diverse activities in the Type strain of this ruminal species.

The three most active isolates demonstrated polysaccharidase activities against α-glucans, β-glucans, cello-oligosaccharides and alginate and there was evidence that activity was induced when growth was on a complex substrate (*L. hyperborea* extract). The possibility exists that other enzymes or higher activities may be detected when the organisms encounter the appropriate polysaccharidic compounds as exemplified by the effect of laminarin on strains L7 and L10.

In the serial culture studies on the whole rumen microbiota only about 40% of the available carbohydrate was removed in the fucoidan supplemented cultures. Although the utilization of this particular polymer was less extensive, the usage was consistent over the period confirming that the ability to degrade this sulfated polymer does exist in the ruminal population and can be maintained in serial culture. However, no single fucoidan degrading isolates were able to be selected on agar plates. Novel strategies may therefore be required to isolate the bacterial species involved in fucoidan breakdown. The failure to isolate the species involved is probably associated with their relative ability to compete in the isolation procedures with organisms in the secondary scavenging population or may indicate dependent syntrophy among two or more species.

The utilization of the seaweed storage carbohydrate mannitol was particularly stable over the period of serial culture. The presence of mannitol utilizing bacteria in the rumen is more specific to animals consuming seaweed as many of the major species of ruminal bacteria do not utilize this sugar alcohol (Stewart *et al*., 1997). Although the identity of the bacteria involved has yet to be established, Orpin and colleagues (1985) observed a much higher proportion of mannitol utilizers in the ruminal contents of North Ronaldsay sheep consuming seaweed than in animals of the same breed fed grass.

That the cultures actively produced methane over the course of the trial confirmed that frozen rumen contents from seaweed-eating sheep are potentially suitable inocula for AD systems and further investigation to optimize the process could result in increased methane yields. Although the proportion of methane produced from the different substrates during serial culture was similar it cannot be assumed that the species profiles were the same. Additional studies are required to identify the microbiological composition of sequential cultures, which is likely to be substrate-dependent.

All serial cultures were acetogenic and accumulated acetate during each 7-day culture period. Acetate is a substrate for acetoclastic methanogenesis.

In conclusion these rumen microbial consortia, established by serial culture, have the potential to be inocula for AD to enhance the utilization of specific seaweed varieties. Alternatively, the individual bacterial isolates recovered (such as L7, L10 and A11) may offer sources of enzymes or may act as adjunct cultures for the efficient AD of seaweeds in renewable biofuel production.

## Experimental procedures

### Collection of rumen contents

Three North Ronaldsay seaweed-eating, mature male sheep were transported to the abattoir at Kirkwall, Orkney. The rumen contents were recovered immediately after slaughter and were combined in a heavy duty polythene bag surrounded with ice packs for transfer to the laboratory in less than 24 h at below 10°C. The pooled rumen contents were divided into completely filled 100 ml screw-capped containers and stored at −20°C.

### Collection of seaweed

Seaweed samples were collected from the inter-tidal area of a beach close to Kirkwall; the sample collected was a mixture of *Fucus* spp. (*F. vesiculosus* and *F. serratus*). Dried samples of *L. hyperborea* and *A. nodosum* were also generously provided by R. McBride (FMC Biopolymers, Girvan) and these seaweeds were stored at −20°C.

### Isolation of seaweed polysaccharide degrading microorganisms from the ruminal contents of seaweed-eating sheep

All media and reagents were prepared using rigorous anaerobic techniques (Stewart *et al*., 1997), including the use of an anaerobic cabinet (Don Whitley Scientific, UK). A basal medium was based on the media described by Caldwell and Bryant (1966). Liquid cultures were made under a CO_2_ atmosphere in glass vials sealed with butyl rubber stoppers and crimped aluminium caps. Media in which the redox potential had not attained −50 mV, and therefore exhibited a pink-purple resazurin indicator coloration, were not used.

The principal seaweed polysaccharides added to the basal medium and used for bacterial enrichment and isolation were alginic acid (5 g l^−1^), laminarin (2 g l^−1^), fucoidan (1 g l^−1^) and cellulose filter paper strips (10 g l^−1^). Dried *A. nodosum* and *L. hyperborea* homogenates were also used at 10 g l^−1^. Agar media were made by adding agar powder (15 g l^−1^). Thawed North Ronaldsay sheep rumen contents were added to the enrichment media as a 1% inoculum and incubated anaerobically at 39°C for 96 h. After enrichment, cultures were serially diluted in anaerobic maximum recovery diluent (MRD) (Oxoid, UK) and 200 μl volumes of the dilutions were spread on agar media containing the same isolation polysaccharide as was used in the enrichment broth. The cellulosic recovery substrate used was carboxymethyl cellulose (CMC, 5 g l^−1^). Plates were incubated anaerobically at 39°C for 48 h.

Recovered colony isolates were inoculated into broths and agar slopes of a maintenance medium consisting of basal medium with 1 g l^−1^ each of glucose and cellobiose.

### Polysaccharide utilization by ruminal bacterial isolates and strain selection

The ability of 65 bacterial isolates to degrade their enrichment culture seaweed- polysaccharide was examined in duplicate 5 ml of broth culture by measuring residual carbohydrate (Dubois *et al*., 1956) and reducing sugars (Lever, 1972; 1977) respectively.

In further studies the range of polysaccharides (alginic acid, laminarin, fucoidan, CMC, at 2 mg ml^−1^) or seaweed extract utilized by nine selected polysaccharide degrading isolates was assessed in broth cultures by measuring residual carbohydrate levels (Dubois *et al*., 1956). Extracts were prepared from dried preparations of *A. nodosum* and *L. hyperborea* and from frozen *Fucus* spp. biomass. The carbohydrate content of extracts, determined by the method of Dubois and colleagues (1956), were 2.9, 3.35 and 2.25 mg ml^−1^ respectively. The media (5 ml volumes) were inoculated with 50 μl of 24 h cultures grown on their initial isolation polysaccharide; except C8 which was grown on laminarin.

### Glycoside hydrolase and polysaccharide depolymerase profiles of selected seaweed polysaccharide utilizing isolates

Enzyme activity profiles were determined in cultures of the three most active isolates grown on the polysaccharide on which they had been isolated originally or on seaweed extract to determine whether a more complex substrate impacted on the activity profile. Duplicate cultures were set up with either alginate (isolate A11) or laminarin (isolates L7, L10) as the carbohydrate substrate at an initial concentration of 2 mg ml^−1^ and triplicate cultures of each isolate with *L. hyperborea* extract (1.83 mg ml^−1^ soluble carbohydrate level). Crude enzyme preparations were prepared by centrifuging and then sonicating bacterial pellets combined from each of the replicate 100 ml of cultures.

Glycoside hydrolase activities were determined by measuring the rate of *p-*nitrophenol release (determined as absorbance at 415 nm) from the appropriate *p*-nitrophenyl glycoside derivatives (5 mM in 0.1 M MES buffer pH 6.5) after incubation for a maximum period of 90 min at 39°C (Williams and Withers, 1982a). Polysaccharidase activity was detected as reducing sugar released after incubation at 39°C for 60 min and quantified in a two-stage assay procedure (Williams and Withers, 1982b). Activities were determined by assay of single samples using enzyme preparations obtained from combined duplicate or triplicate cultures.

### Bacterial identification to species level by molecular characterization

DNA was extracted from bacterial isolates by boiling individual colonies from agar plate cultures (for 15 min in 1 ml of distilled water) and then removing contaminant protein by precipitation with Chelex resin (Bio-Rad Labs, UK). The DNA was PCR-amplified with primers specific to the 16S rRNA gene by the method and primers of Tajima and colleagues (1999). Primers were: 27f- 5′-AGAGTTTGATCCTGGCTCAG-3′ and 1544r-5′-AGAAAGGAGGTGATCCAGCC-3′.

The resultant PCR amplicons were sequenced at the Dundee Sequencing Laboratory (College of Life Sciences, University of Dundee, Scotland) and the sequences derived were blast searched for homology with 16S rRNA gene library sequences at http://www.ncbi.nlm.nih.gov.

### Serial culture of whole rumen microbiota

Cultures (5 ml volumes) were in single seaweed polysaccharide or whole-seaweed homogenate supplemented basal medium initially inoculated with homogenized ruminal contents that had been stored prior to use at −20°C. After incubation at 39°C for 7-day a follow-on serial culture was set up using a 10% inoculum from the established culture. The substrates used (2 mg ml^−1^) were alginate, laminarin, fucoidan, carrageenan, cellulose (filter paper), amylopectin, xylan and mannitol. Cultures were also set up using *L. hyperborea, A. nodosum* and *Fucus* spp. homogenates at 10 mg ml^−1^. The residual carbohydrate at the end of each 7-day serial culture period was determined by the method of Dubois and colleagues (1956).

### Analytical techniques

Headspace gases in serial cultures were analysed by gas chromatography using a Perkin Elmer 8500 Gas Chromatograph fitted with a methanizer and flame ionization detection. A 6 feet × 1/8 inches column packed with 80/100 carbosphere was used with a helium carrier gas flow rate of 20 ml min^−1^ (Speck and Burke, UK). The separation was run isothermally with an oven and injector temperature of 150°C; the detector temperature was maintained at 300°C. Peak area was calculated with a Hewlett Packard HP3396 series II in-line integrator.

Cell-free culture supernatants were analysed for the presence of acetate using an enzyme assay kit (R-Biopharm). The method was adapted for use at the 96-well microtitre plate scale by reducing reagent/sample size by a factor of 10. The analytical method and calculation used were as described by the manufacturer. A standard curve comprising 0–2.96 μg of acetate in the assay system was constructed for each assay. The basal medium contained approximately 1.7–1.8 mg ml^−1^ acetate and due allowance was made for this endogenous component in determining assay results.
